# Challenges and Advances in Peripheral Nerve Tissue Engineering Critical Factors Affecting Nerve Regeneration

**DOI:** 10.1155/2024/8868411

**Published:** 2024-09-11

**Authors:** Massoumeh Jabbari Fakhr, Fatemeh Kavakebian, Shima Ababzadeh, Alireza Rezapour

**Affiliations:** ^1^ Department of Tissue Engineering and Applied Cell Sciences School of Medicine Qom University of Medical Sciences, Qom, Iran; ^2^ Cellular and Molecular Research Centre Qom University of Medical Sciences, Qom, Iran

## Abstract

Peripheral neuropathy is painful and can cause a considerable decline in quality of life. Surgery and autograft are the current approaches and clinical standards for restoring function after nerve damage. However, they usually result in unacceptable clinical results, so we need modern peripheral nerve defect treatment approaches. Tissue engineering techniques have been developed as a promising approach, but there are some considerations for translational application. Clinical application of novel tissue engineering methods is related to combining the appropriate cell and scaffold type to introduce safe and efficient bioscaffolds. Efficient nerve regeneration occurs by mimicking the extracellular matrix and combining topographical, biochemical, mechanical, and conductive signs via different cells, biomolecules, and polymers. In brief, ideal engineered biomaterial scaffolds will have to cover all characteristics of nerve tissue, such as nerve number, myelin, and axon thickness. Nerve regeneration has a highly sensitive response to its surrounding microenvironment. For designing a suitable construct, matching the regenerative potential of the autograft as the golden standard is essential. This review article examines the newest advancements in peripheral nerve tissue engineering. Specifically, the discussion will focus on incorporating innovative cues, biological modification, biomaterials, techniques, and concepts in this area of research.

## 1. Introduction

A peripheral nerve is injured due to different events such as diabetes, immunologic reactions, and physical damage, affecting millions worldwide and leading to social and economic burdens. An exciting feature of the peripheral nervous system (PNS) is the ability to regenerate after injury. The efficacy of spontaneous regeneration depends on the extent of the damage incurred and endogenous neuronal signals, and the lack of any external intervention is unlikely to result in an acceptable functional result [1].

In cases where the body cannot compensate for and repair severe nerve damage, the potential for improvement may be permanently limited. For designing alternative therapy in neuropathy, native architecture containing the surrounding microenvironment, surface topography, biochemical signals, electrical activity, axonal guidance, extension and neurotrophic factors (NFs), and extracellular matrix (ECM) proteins must be considered. Nerve regeneration is very complex, so many approaches have been used to solve this complexity [2].

The field of tissue engineering offers a promising path for developing complex structures that can be utilized in translational medicine [3]. An examined approach for neurotherapy involves using natural or synthetic nerve guidance conduits (NGCs). Engineered NGCs were varied in content (biomaterials, cell, growth factor, exosome [4], etc.), fabrication method, and especially in functional outcomes [2].

Autografts are still the most effective method for managing peripheral nerve injuries despite the need for donor tissue. Thus far, the implementation of biomaterials and manufacturing techniques has not matched autografts as the most effective method in treating critical-sized nerve defects [2, 5]. An engineered construct is needed to stimulate neural survival and facilitate axon extension and guidance to achieve optimal neurologic function, reduce recovery time compared to autografts, and facilitate axon extension and guidance [2]. However, guiding the proliferation, differentiation, and transplanted replacement or supporting cells is only a fraction of the challenges that must be overcome in tissue engineering-based design and evaluation. Here, we describe the methods for generating peripheral nerve constructs and strategies for cell, biomaterial, and other cue selection.

## 2. Neural Development Native Tissues

### 2.1. The Construct of Peripheral Neural Tissue

The peripheral nervous system consists of nerves (specifically, spinal, cranial, and visceral nerves) and ganglia, which act as conduits for transmitting nerve impulses and nutrients to neurons. The neuron, the structural unit of the nervous tissue, consists of a nerve cell body and several processes: dendrites, axons, and axon terminal.

The primary focus of experimental investigation in peripheral nerves is centered on the aggregation of axonal bundles, comprising a heterogeneous combination of myelinated and unmyelinated axons, that conduct efferent (motor) or afferent (sensory) electrical signals. Schwann cells (SCs), satellite cells, enteric glia, and olfactory ensheathing are the primary glial cells in the peripheral nerve tissue. SCs generate Bungner bands, which are elongated tubular fascicles [3]. A total of 43 nerves, including 12 pairs of cranial nerves, 31 pairs of spinal nerves, and autonomous nerves, form the foundation of the peripheral nervous system. Most studies and clinical trials on peripheral nervous repair focus on the brachial plexus and sciatic nerve [6].

### 2.2. Microenvironment

Improved understanding of nerve regeneration trends leads to the development of better approaches for regeneration. Native tissue features are closely related to the strategies employed for neural constructs. So, clarifying every tissue's biological, physicochemical, and mechanical properties is critical for designing an appropriate scaffold. Any engineered structure must mimic the complicated native architecture and physiology. The biomimicking of the nervous tissue microenvironment requires neurons and diverse types of glial cells dispersed across multiple nervous system regions. This cell population's density, diversity, and interactions determined tissue phenotypes. The 3D environment of cells is a critical factor that significantly affects the functionality of native tissues. The dynamic microenvironment, cell-cell, and cell-ECM connections are directly related to the developmental and physiological events of native neural tissue [6].

Nervous ECM differs from other organs as it supports cells and modulates their behavior. In nervous tissue ECM, lecticans, hyaluronan, and tenascins dominate; unlike other tissues, collagen, fibronectin, and laminin are rare [7]. ECM molecules consisting of growth factors, cytokines, and chemokines are the other critical neurotrophic factors that promote neural development and regenerate through concentration-dependent gradients [7, 8]; the spontaneous repair mechanism in the peripheral nerve results in poor reinnervation after a pathophysiological event. After a peripheral nerve injury, irreversible pathophysiological changes lead to an imbalance in the neuronal microenvironment, resulting in scar tissue formation and poor functional restoration [9]. Wallerian degeneration, a procedure that considered axon degeneration and myelin failure, is known to occur in the distal end. Concurrently, axonal degeneration occurs in a small distal zone to the proximal stump. Ranvier nodes are the origin of axonal sprouts that Schwann cells can myelinate [10]. Failure of the regenerative environment because of loss of trophic support and growth factors such as glial cell-derived growth factors (GDNF) and brain-derived neurotrophic factor (BDNF) and increase in growth-inhibiting molecules such as chondroitin sulfate proteoglycans in the distal axonal segments was causing the failure of reinnervation [3]. SCs are highly mobile and can migrate toward damaged tissue, which is crucial in regenerative tissue. Moreover, they secrete signaling molecules that attract macrophages, facilitating neuronal survival, promoting axon growth, activating resident mesenchymal stem cells, and interacting with various other cell types [11]. The support structures provide neurotrophic support to affected areas, restrict the penetration of fibrous tissue, and direct the growth of regenerating axons towards their appropriate targets [12].

After transplantation in the body, any tissue engineering construct would trigger a response from the body. The reaction to scaffolds is intricate since it involves the removal of migrated macrophages and monocytes from myelin and axon debris. On the other hand, activating inflammatory cells triggers the secretion of specific factors that promote creating a regenerative environment. The regeneration of peripheral nerves is influenced by several factors, such as the selection of biomaterial, the fabrication technique used, the rate and byproducts of degradation, and their effect on the regenerative microenvironment and their interaction with nerve cells. These factors can impact the neural microenvironment significantly [13]. Scaffolds and host cells/tissues are affected by each other. Therefore, it is desirable to have a construct that can change the direction of tissue repair.

The regeneration of peripheral nerves depends on a well-balanced local microenvironment. Degradation products of scaffolds, mechano- and electrotransduction properties, degree of tissue inflammation, and immune and cellular functions are crucial for regenerating peripheral nerves. Also, elements such as Schwann cells, polymorphonuclear cells, lymphocytes, plasma cells, giant cells, vascular endothelial cells, fibroblasts, macrophages, and biomaterials are involved in the local regenerative microenvironment and inflammatory and immune response. Overall, the microenvironment of neural regeneration is influenced by five crucial components: immune response, intraneural vascularization, bioenergetic metabolism, and biomechanical and bioelectrical conductions and their signaling pathways. Each of these components plays a crucial role in promoting the restoration of neural tissue [9, 13].

The polarization of macrophages from a proinflammatory state to an anti-inflammatory state, as well as changes in tissue structure such as necrosis, fibrosis, and fat infiltration, facilitate the promotion of peripheral nerve regeneration [13]. The support structures play a significant role in providing neurotrophic support to the affected regions while limiting the infiltration of fibrous tissue and directing the growth of regenerating axons towards their targets [11]. The conversion of mechanosensing stimuli into biochemical signals induces adaption in response to their surrounding microenvironment [14].

The microenvironment is crucial for determining and facilitating nerve repair cascade by integrating coordinated signals. The cell signaling activated by the extracellular matrix molecules functions to potentiate the role of the matrix in repairing extended peripheral nerve injury [15].

In the context of injury, SCs serve as myelinating cells. These cells can undergo dedifferentiation through the activation of TGF*β* signaling, resulting in the gaining of mesenchymal characteristics and a transition to a progenitor-like state [16].

## 3. Engineering Approaches for Nerve Repair

Treatment approaches combining cell transplantation, molecule delivery, and biomaterial scaffolding will provide hope for tissue regeneration, repair, replacement, and efficient recovery. Developing and advancing NGC design, biomaterials, fabrication methods, cellular compounds, biomolecules, fillers, and topological cues will offer feasible neuropathy treatments.

### 3.1. Cell-Based Techniques

Cellular components are essential for combined therapy and scaffold efficiency in nerve injury healing involving multiple cell types. In cell-based therapy, selecting cell sources, administration routes, dosages, fates, and survival are significant challenges. It is worth noting that including SCs in autologous grafts plays a critical role in providing neurotrophic support and ultimately determining the positive outcomes of autografts in cases of motor nerve damage. Therefore, the significance of cellular selection is essential in cell-based therapy for nerve injury healing [3]. Previous studies have found cell selection for seeding sources very challenging. Regardless of the specific cell type, various autologous, allogeneic, and xenogeneic sources have been previously employed in the field of neural tissue engineering. The process of obtaining and collecting autologous neural cells and managing the immunological reactions of allogeneic cells has become challenging. Numerous factors must be considered when selecting a cell source, including limited resources, accessibility, purification, immune reactions, pathogen contamination, large-scale expansion, ease of harvesting, and tumorigenic and ethical issues.

Various cell populations play a vital role in the construction of tissue engineering for the peripheral nervous system. In particular, neuronal progenitor cells [17], neural stem cells [18], neurons, SCs [19], olfactory ensheathing cells (OECs) [20, 21], satellite glial cells (SGCs), and enteric glial cells (EGCs) were utilized (Table 1). Each cell type possesses unique characteristics that contribute to the tissue engineering process and are critical to successful outcomes. Research indicates that stem cells support axon regeneration using trophic mechanisms that influence SCs and increase angiogenesis [19].

**Figure 1:**
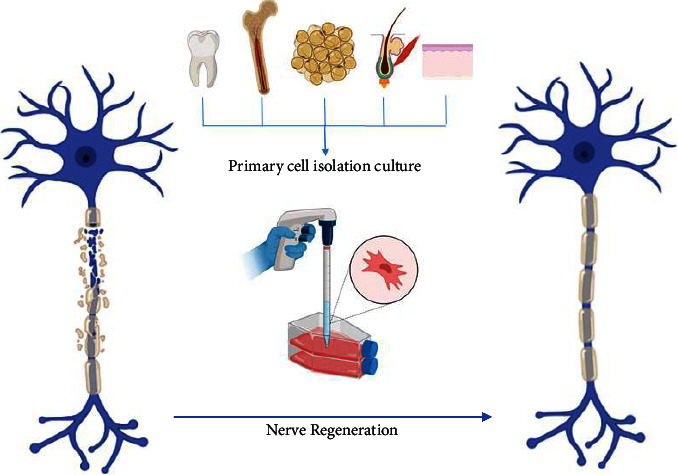
Schematic representation of sources of cells seeded in peripheral nerve regeneration.

Different sources of stem cells have been used in regenerative therapy, including embryonic stem cells [17], bone marrow stromal cells (BMSCs) [18], adipose-derived mesenchymal stem cells (ADMSCs) [20, 29], umbilical cord mesenchymal stem cells [21], hair follicle stem cells [30–32], dental pulp stem cells [33], skin-derived precursor cells (SKPCs) [34], and induced pluripotent stem cells (iPSCs) [33, 35, 36] and even dorsal root ganglion (DRG) [37], fibroblast [38], and immunomodulatory cell have been used previously in regenerative therapy (Figure 1). The effectiveness and viability of supportive cell implantation in nerve conduits require further investigation to gain a comprehensive understanding. The current research indicates that there is still uncertainty in this regard [38]. Stem cells' secretion of neurotrophic factors and exosomes suggests that they can differentiate into Schwann cell-like (SC-like) cells that facilitate peripheral nerve growth and myelination growth [39].

After cell source selection, cell coculture, gene-modified stem cells, engineered SCs [39], and suitable materials for 2D and 3D cell culture that can improve different cell populations' viability and functional integrity are very discussable. Biological constructs were formed *in vitro* by coculturing DRGs and SCs of multiple types [10, 40]. The scaffold's biological activity was improved in the different studies via gene-modified stem cells used for cell-based expression and delivery of nerve growth factor (NGF) [41], neurotrophin-3 (NT-3) [42], GDNF [43], and BDNF [44, 45].

SCs are an essential component of the healing process in the PNS. SCs are integral parts of regeneration that can differentiate from MSCs derived from bone marrow, adipose, and dental pulp. MSCs possess excellent differentiation capacity and proliferation potential, which enables them to transform into SC-like phenotypes and promote nerve remodeling [2, 12, 46, 47]. This ability has many clinical benefits and advantages. After MSCs differentiate into SC-like, these cells organize bands of Bungner, axon regeneration, and myelination. Growth factors then stimulate regeneration and ECM secretion via paracrine signaling. After selecting the appropriate target cell for nerve repair, the niche of those target cells will be provided via molecular or mechanical stimulation or both [6].

Although the precise mechanisms underlying the therapeutic benefits of MSCs remain fully elucidated, existing evidence suggests that these cells can promote anti-inflammatory and immune regulatory effects in the context of peripheral nerve injury. Furthermore, MSCs can attenuate muscle mass loss while altering the microenvironment towards a proregenerative phenotype [48]. The remarkable regenerative capacity of the peripheral nervous system is attributed to the inherent plasticity displayed by SCs. In the aftermath of PNS damage, SCs undergo a dedifferentiation process and transition into repair phenotypes. These phenotypes are instrumental in promoting axonal regeneration, facilitating myelin formation, and disposing of axonal and myelin debris. Supplementing necrotic regions with SCs via gene modification or stem cell transplantation is clinically valuable due to the restricted self-repair capacity of SCs for extended peripheral nerve defects. Furthermore, developing tissue-engineered nerves with bioactive scaffolds represents a promising strategy with great potential in addressing such tissue defects [39].

### 3.2. Bioactive Molecules Supplementation

The inclusion of biochemical components in neural scaffold architecture presents a significant challenge. Numerous barriers must be overcome to ensure such components' successful integration and subsequent efficacy [6, 49]. Growth factors' short half-life and rapid degradation within body fluids make administering them directly to the distal end challenging [50, 51]. So, adding exogenous growth-promoting agents within the ECM proteins of the interior of the NGC is vital for efficient deployment and continuous local delivery. These agents are comprised of a diverse range of small molecules, growth factors, cytokines, biochemical cues, adhesion molecules, signaling factors, inflammatory mediators, antioxidant reagents, bioactive peptides, and ECM protein that have specified enhancement effects on the axonal regeneration and functional outcomes [6].

After an injury, endogenous growth factors released from the distal end of the nerve can regulate axon regeneration. It is noteworthy, however, that exogenous growth factors may need to be present continuously to stimulate the repair process. Axonal regeneration is facilitated by aligned SCs and their ECM, which are made of laminin and fibronectin and provide vital pathways and growth factors [3].

Appropriate cell and extracellular matrix interactions are critical in tissue-engineered designs. Engineered microenvironments and scaffold modification using natural or non-native growth factors, biomolecules, or peptide gradients can potentially improve this interaction. Incorporating bioactive peptides into a scaffold through a biofunctional epitope significantly enhances cellular behavior, including adhesion, proliferation, migration, differentiation, and alignment. This process can be achieved by utilizing various biomolecules and methodologies.

Researchers have specifically explored sequences that are relevant to neural adhesion. These include the RGD peptide found in collagen, laminin, and fibronectin. Peptides derived from laminin consist of YIGSR, IKVAV, RNIAEIIKDI, and RYVVLPR. Polylysine and various proteins such as integrin, talin, vinculin, *α*-actinin, filamin, and paxillin are also included. Modifying biomaterial surfaces with cell adhesion molecules involves utilizing several methodologies such as blending, surface deposition, electrostatic, and covalent attachment. The selection of appropriate adhesion molecules, their coatings' stability, and their optimal concentration are paramount in promoting axon repair [52–54].

Promoting axon repair through growth factors induces early regeneration, increasing nerve growth near the injury site. However, it is essential to note that the increased growth factors are not sustained indefinitely, as they eventually decrease to prevent the immune system from attacking the regenerating axon. Ultimately, successful innervation is achieved through this process [47]. Any of the cellular sources described previously can initiate growth factor generation.

**Figure 2:**
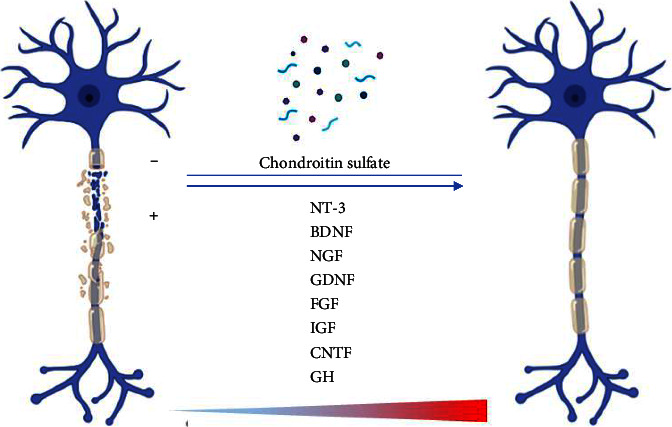
Schematic illustration of the application of growth factors in treating peripheral nerve injuries.

Several growth factors are commonly used for neural tissue engineering, which can be classified into two categories: (1) neurotrophins and (2) neurotrophic action factors. The first category includes neurotrophin-3 (NT-3), BDNF, and NGF. The second category comprises GDNF, endothelial cell factors, fibroblast growth factors (FGFs), insulin-like growth factor (IGF), ciliary neurotrophic factor (CNTF), systemic growth hormone (GH), or a combination of these factors. Platelet-derived growth factors (PDGFs) have been used as filling agents for nerve conduits and as scaffolds for nerve stumps [55–57] (Figure 2).

SCs, the critical factor in nerve regeneration, can secrete neurotrophic factors, N-cadherin, neurotrophins, gamma integrins, neural cell adhesion molecules (N-CAMs), and collagen and laminin [58]. The existence of ECM proteins, including collagen I, collagen IV, laminin, fibronectin, and neurite guidance proteins, strengthens the tissue regeneration process. Integrins play a crucial role in facilitating axonal regeneration through their ability to interact with key signaling ligands. Recent studies have shown that laminin and integrin's “outside-in” signaling is critical in nerve regeneration. It has been determined that the laminin-binding domains (LBDs) are necessary components for proper BDNF and CNTF functioning [59].

The process of transdifferentiating stem cells into a Schwann cell-like phenotype involves exposure to various agents. These agents include *β*-mercaptoethanol, all-trans retinoic acid, fetal bovine serum, forskolin, recombinant human bFGF, recombinant human platelet-derived growth factor-AA, recombinant human heregulin *β*-1, and glial growth factor [60, 61]. The gradient of granulocyte colony-stimulating factor (G-CSF) displays significant potential for neurotrophic and angiogenic effects [62]. Bioactive agents' precise delivery and control are crucial for repair processes and can affect the three-dimensional cell orientation. In this regard, a multitude of axone-promoting cue delivery systems is available, including distribution on scaffold surfaces, attachment to scaffold walls, incorporation, lumen fillings using hydrogel, microspheres, covalent immobilization, and osmotic minipump or injection device-supported continuous release. Bioactive three-dimensional scaffolds are considered a suitable substrate for administering axon-promoting cues that can effectively enhance the process of nerve regeneration. Gene-modified SCs or stem cells can express various growth factors, including FGF, BDNF, CNF, and GDNF, which can be used for cell-based delivery [45]. Previous research has demonstrated this approach through transduced SCs [41, 43]. Modification of neurotrophic factors is an approach that can be employed to sustain their activity during elongated release from the cellular environment. Immobilization techniques, such as crosslinking or photochemical reactions, as well as coaxial electrospinning, have been utilized for this purpose [63]. Furthermore, it has been determined that their capacity to bind with cells or scaffold biomaterials at a high-affinity level presents a feasible resolution.

Another method for enhancing nerve regeneration involves differential adsorption of growth factors into neural scaffolds [2]. Recent research has identified a significant biomolecule known as the stem cell recruitment factor, demonstrating the ability to enhance the functional regeneration capacity of host stem cells. Specifically, substance P (SP) has been found to attract stem cells and modify NGCs through a micropattern-based fabrication method. These findings highlight the potential of SP to play a critical role in neural tissue engineering [64].

Now that we have investigated the intricate world of bioactive molecules and their pivotal roles in cellular processes, it is critical to shift our attention to the structural scaffolds. The successful integration and sustained delivery of bioactive components within the neural guidance channels pose significant challenges, necessitating a thoughtful consideration of the scaffold's structural design. The synergy between bioactive molecules and scaffold architecture is vital to coordinating precise cellular responses and tissue regeneration. Therefore, in the following, we investigate how different generations of scaffold designs, including different luminal fillers, affect the functionality and performance of these essential biomaterials in the field of nerve regeneration.

### 3.3. Scaffold Configuration

The configuration of scaffolds significantly impacts the topological properties and cell fate of neural guidance channels, ultimately influencing the performance of neural scaffolds [49]. Three generations have been established based on luminal fillers to classify NGCs. The architecture of peripheral nerves depends on the structure of elongated tubular fascicles composed of Bungner fibers. The first generation of these fibers is characterized by a simple hollow tubular duct without any topological indication. To accurately mimic the endoneurium in nerve structure, it is necessary to incorporate intraluminal microarchitecture and account for the frequently porous nature of the external wall [47, 65]. Thus, the next generations and more complex types of NGC were created. The fillers comprise fibers, filaments, microchannels [66], gels, sponges, and grooves [6, 65, 67]. A compact filler could potentially inhibit the development of neural pathways [5].

## 4. Structural Characteristics of Scaffolds

The physical properties of the scaffold are related to the material and method of fabrication. The nerve scaffold can be constructed using synthetic, biological, or hybrid materials, designed into different morphologies such as tubular, fibrous, and matrix types [2, 68]. Three significant factors are highlighted here as follows:

1. Porous conduit and its pore size profoundly impact the exchange of nutrients and growth factors, cell viability, adverse cell invasion, and vascularization [69].
2. The impact of the scaffold's flexibility on the compression and mechanical strength of the conduit is notable, as it inhibits any harm to the regenerative tissue [2, 70].
3. Speed of degradation has a significant impact on the processes of inflammation and swelling, as well as tissue compression. Furthermore, this velocity also regulates regeneration and prevents inflammation and compression [2, 6].

### 4.1. Porous NGCs

The three-dimensional porous structure has a significant impact on the NGC's properties. The pores are designed to facilitate the migration of cells, ensure a sufficient supply of nutrients and oxygen, and remove metabolic byproducts. Regarding size, permeability, structure, and connectivity, the pore architecture within the scaffold needs to align with the pathophysiological requirements for repairing peripheral nerves by considering the synergistic effects of various cells and bioactive factors. Pore dimensions and structure impact cellular responses, such as adhesion, expansion, and migration. The permeability of the pore structure is closely associated with its beneficial function in facilitating the regeneration of peripheral nerves. Also, porosity is crucial in influencing the surface-to-volume ratio, mechanical characteristics, and biodegradation rate of NGCs. Various fabrication techniques exist for each NGC morphology to develop innovative pathways and create the most appropriate structure and topography conducive to optimal neural growth [71, 72].

### 4.2. Fabrication Techniques

Neural scaffolds can be fabricated through various conventional microfabrication techniques, such as immersion precipitation particulate leaching [73], extrusion [74], injection molding solvent evaporation [75], nonwoven or woven mesh rolling [76], centrifugal casting [76], spinning mandrel technology [77], electrostatic direct writing [78], cutting holes into the walls, film casting plus rolling, and molding plus freeze-drying [60]. In addition, advanced techniques such as electrospinning of fibers [61], phase separation [79], gas foaming [80], gradient freezing method, in situ polymerization [63], self-assembly [81], micropattern [82], computer-aided design-based fabrication techniques [66], and bioprinting can also be utilized (Table 2).

Regardless of the method employed in construction, the characteristics of the structure hold considerable significance in ensuring favorable clinical outcomes. The construct's intricate internal architecture and topology impact various factors, including cell adhesion, proliferation, and differentiation. This effect is observed both on the surface and within the scaffold. In addition, it influences biodegradability, biocompatibility, controlled degradation, reabsorption of metabolites, immunogenicity, and mechanotransduction [46, 94].

### 4.3. Biomaterial Selection

A scaffold is a substrate for the adhesion, proliferation, migration, and function of neural companion cells. A range of synthetic and natural polymers can create an ideal scaffold [2, 6]. The biomaterial selection for scaffold fabrication depends on the scaffolding's expected function [1]. For ideal neural scaffold fabrication, selecting biomaterials that align with biological, biomechanical, and physicochemical considerations is crucial. Appropriate properties are usually obtained by combination or modification [6].

Biomaterials should meet specific criteria that ensure they are safe for use in the body. They should not be toxic, allergenic, mutagenic, or carcinogenic. In addition, they should minimize scar tissue formation and promote the growth and recovery of target cells. They should also be capable of controlling mass transport and providing biological factors that aid in cell adhesion, proliferation, survival, and differentiation. Selecting appropriate materials for neural tissue engineering techniques, particularly bioprinting, has become increasingly complicated. A limited selection of materials can provide the necessary characteristics of printability, cell support, and mechanical reinforcement. Generally, hydrogels have emerged as a suitable choice for bioprinting applications [1].

The position of exogenous or endogenous cells, cellular adhesion, the formation of the ECM, and the general improvement of repair are related to the type and characteristics of the material incorporated into the scaffold composition. Some biomaterials can be intentionally engineered to provide detectable microenvironmental cues, resulting in modifying the phenotype of a cell, for example, the changing polarization of invasive macrophages into a regenerative phenotype [95]. The mechanical strength and flexibility properties of synthetic materials, especially conductive polymers, have caused a significant increase in the extension and proliferation of neurites [46].

All aspects of scaffold fabrication strategies must be considered, including porosity, permeability, flexibility, stiffness, biological features, biocompatibility, biodegradability, conductivity, surface area, and properties [96]. A critical characteristic of a suitable scaffold is its ability to support cellular function and mimic the natural ECM [1]. The neural scaffold can distribute biochemical agents via diffusion [10]. The conduit's porosity promotes angiogenesis and inhibits scar tissue [97]. The scaffold's biocompatibility is critical in facilitating cell adhesion, proliferation, migration, and maturation.

It is worth noting that unwanted bodily reactions are often experienced, and inducing inflammation may produce positive and negative effects on the regeneration of the peripheral nervous system. As shown in Table 1, a wide range of scaffolds of different categories, including natural, synthetic, and nanofibrous variations, has been extensively evaluated for their potential use in the field of neural tissue engineering.

### 4.4. Conductive Biomaterials and Electrical Stimulation

The ability of neurons to produce electrochemical signals is a fundamental aspect of the nervous system. Several investigations have shown that electrical excitation has the potential to facilitate nerve regrowth [98, 99]. This method has typical clinical applications but has limitations for deep muscles, which the tissue engineering approach resolves. As previously detailed, a fundamental attribute of appropriate materials for producing nerve tissue scaffolds is their electrical conductivity. Materials that exhibit electrical conductivity and piezoelectric properties are categorized as electrically conductive materials. Examples of such conductive materials include polypyrrole (PPy), polyaniline (PANI), polythiophene, polyphenylene sulfide, conductive nanoparticles, such as gold and silver nanoparticles, graphene nanosheets, carbon nanotubes, poly(3,4-ethylenedioxythiophene) (PEDOT), and nanoparticle [100]. Also, piezoelectric materials include black phosphorus and piezoceramics [101].

The effects of electrical conductivity on cellular neurite outgrowth, cell division, cell polarity, differentiation, migration, and angiogenesis have been investigated regarding their patterns, strengths, and time courses [2]. Through electrical stimulation within conductive scaffolds, effector proteins such as BDNF, NGF, CNTF, vascular endothelial growth factor (VEGF), and calcium ion channels can be activated. This activation subsequently increases cAMP levels, leading to the extension of neurites, nerves, and myelinated fibers and, ultimately, an elevation in the repair of both motor and sensory nerves in the direction of the administered electrical current [102]. It is imperative to acknowledge that the critical function of neurons in generating electrochemical signals plays a pivotal role in the innate nervous system. Extensive research has demonstrated that electrical excitation can significantly enhance nerve regrowth [98]. As such, using conductive material becomes essential to stimulate a functional recovery of both motor and sensory pathways and reduce muscle atrophy [47].

Integrating conductive biomaterials and electrical stimulation presents significant potential for advancing nerve tissue engineering. Conductive NGCs can produce electrical energy by themselves, making them an excellent option for self-powered nerve scaffolds. This approach offers several advantages, including enhanced nerve regrowth, precise control of cellular processes, and functional recovery of both motor and sensory pathways. However, successfully translating this technology into clinical applications depends on addressing critical challenges related to biocompatibility, optimal stimulation parameters, and integration with other scaffold components [103, 104].

### 4.5. Traditional Biomaterials Selection

The utilization of autografts for nerve regeneration is subject to limitations, including the inconsistent thickness of regenerated nerves [78]. Using commercially available artificial NGCs is a viable solution to this issue, as well as for the other restriction of autograft (Table 3). Neural scaffolds have gained significant outcomes in numerous clinical applications. Multiple research studies have been published focusing on the commercially available items of neural scaffolds and their resultant clinical impact [5].  Table 4 shows that the FDA-approved product structure primarily comprises natural and resorbable materials, with some synthetic and nonresorbable elements included [3]. FDA-approved commercial NGCs are crucial in peripheral nerve repair and regeneration.

Nonetheless, these conduits have certain limitations. The conduits approved by the FDA are unsuitable for the third-generation NGC and do not contain cells, growth factors, or internal patterns or structures. New advanced NGCs consist of multiple layers containing innovative materials supporting cell populations and releasing stimulating factors.

On the other hand, these products are intended for short-distance nerve injuries of less than 3 cm and can only restore nonessential sensory nerves. Up-to-date research is essential to address patient needs and intolerance cases, decrease costs, and develop success rates and industry needs. Researchers are investigating innovative strategies to improve performance, such as complex structures, intraluminal scaffolds, growth factor integration, and autologous and allogenic supporting cells to improve nerve regrowth. Commercial NGCs remain a valuable tool in peripheral nerve repair despite these limitations.

In the clinical usage of NGC, irrespective of nerve diameter and lesion location, choosing a particular conduit causes a challenge due to the lack of actual comparative data among different conduits [105].

## 5. Implantable Scaffolds

The commonly used scaffold in neural tissue engineering is known as NGC, which is typically found in tubular structures, either hollow or solid. The most common forms of these scaffolds are hydrogel and fibrous structures (Table 5).

### 5.1. Hydrogels

Hydrogel-based scaffolds exhibit unique characteristics that make them suitable for regenerating soft tissue. These scaffolds have regulating and unprecedented physicochemical properties, which create 3D-supporting substrates for various cellular processes. Moreover, they establish a hydration network within a three-dimensional space, are injectable [108], and can adapt to irregular injury site patterns of different dimensions and shapes. The printability capacity of hydrogel presents an opportunity to establish hydration networks in three-dimensional space, which can be adapted to various sizes and forms of neural tissues and utilized as substrates for the adhesion, proliferation, and differentiation of neurogenic cells, particularly axons [47, 109, 110]. Furthermore, their application has facilitated the delivery of encapsulated cells, ions [111, 112], bioactive molecules, growth factors [108], nanoparticles [112], and drugs via controlled delivery vehicles in peripheral nerve tissue engineering. Also, hydrogels can transport medicines and growth factors to specific tissue areas with varying concentration gradients.

**Figure 3:**
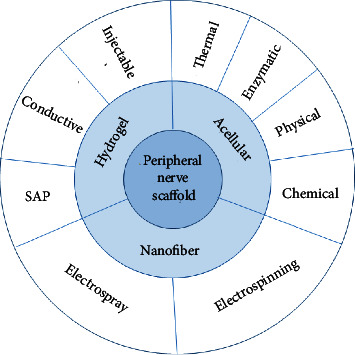
Overview of nerve replacement for engineering neural regeneration.

Among the various biomaterials employed in scaffolding, hydrogels based on naturally occurring biological macromolecules, particularly proteins, polysaccharides, and extracellular matrix hydrogel [113], have demonstrated remarkable outcomes due to their unique physicochemical properties and physiological relevance. The structural attributes of hydrogels can be significantly transformed by environmental factors such as pH, temperature, light intensity, and enzymes, leading to self-assembled peptide (SAP) hydrogels (Figure 3).

### 5.2. Nanofibrous Scaffolds

Advancements in nanomedicine have enabled the regeneration of peripheral nerves using fiber scaffolds at the micro- or nanoscale level, which have become valuable tools in tissue engineering. Fibrous scaffolds are a better alternative to nerve suturing and grafting [2]. However, nanofibers are more effective than microfibers as they promote better cell adhesion, expansion, and proliferation. Nanostructured neural scaffolds have demonstrated promising results in enhancing their bulk and surface properties [6, 114].

The nanoscale surface area, combined with electrical activity, creates an environment that promotes cell adhesion and regulates cell response through the controlled release of bioactive molecules. This property facilitates the regeneration of peripheral nerves through nanofibers and nanoparticles, which also aid in drug delivery [49, 115]. Scaffolds at the nanoscale have the potential to regulate cell cycle progression, cellular viability, and growth factor secretion through surface modifications. These findings significantly affect the field of tissue engineering [9, 115]. For example, electrospinning enables precise control over fiber mechanical properties, size, structural regularity, diversity, fiber orientation, and conduit shape while incorporating various sources such as materials, cells, drugs, and growth factors [116].

Despite the established toxicity of metallic nanoparticles, whether employed individually or in conjunction with scaffolds or conduits, they have demonstrated the ability to manipulate cellular behavior, modify the topography of scaffolds and conduits, increase the secretion of neurotrophic factors, improve ion flow, and regulate electrical signals [117].

### 5.3. Acellularized Scaffolds

Utilizing acellularized ECMs is an effective strategy for making nerve conduits, especially for commercially viable grafts [113, 118]. Acellularized natural tissue can provide a suitable environment for cell growth and proliferation by creating a cell niche [3]. Autologous, allogeneic [119–121], or xenogeneic [119, 122, 123] neural and non-neural tissues were acellularized recently. A notable benefit of this process is the prevention of immune reactions, particularly in xenograft tissue [84, 122, 124].

Utilizing an acellularized ECM has been shown to positively impact the continuous delivery of cells and NFs while inducing the release of NFs such as NGF, GDNF, and BDNF. Furthermore, it has been observed to increase the expression of markers associated with SCs and angiogenesis, specifically VEGF and CD34. Moreover, it promotes axonal regeneration and functional recovery and decreases inhibitory chondroitin sulfate proteoglycans [47, 125–127]. The acellular scaffold exhibits similarities to an autograft by its ability to preserve laminin. This preservation of laminin is a crucial factor in the scaffold's ability to support tissue regeneration.

Acellular nerve grafts have significant drawbacks, such as loss of ECM components and natural ultrastructure. However, these issues can be addressed with optimized multichannel acellular nerve allografts and a modified protocol that preserves more ECM bioactive molecules and regenerative factors while eliminating cellular antigens [128].

Different acellularization techniques, including physical [129], chemical [45], enzymatic, and thermal [130] methods, have been employed to remove cellular components and immunogenic agents. The optimal acellularization protocol should preserve ECM proteins and native nerve architecture. Previous studies recommend implementing an acellularization protocol that prioritizes the preservation of ECM proteins, specifically collagen and laminin while maintaining the native nerve architecture to achieve the most favorable outcome [2, 47]. Multiple studies have demonstrated that using sodium dodecyl sulfate (SDS) [126], Triton X-100, and 3-[(3-cholamidopropyl) dimethylammonio]-propane-1-sulfonate (CHAPS) at the appropriate concentration and duration represents the optimal selection [122] (Figure 3).

## 6. Effect of Scaffolds on Angiogenesis

Improper vascularization frequently leads to tissue necrosis in numerous cases, and neovascularization is necessary for the survival and integration of scaffolds. So, this treatment, with insufficient neovascularization and poor nutrient supply, leads to incomplete functional recovery, especially for significant nerve defects. Research on peripheral nerve scaffolds focuses on enhancing neuronal and axonal repair rather than vascularization. The application of diverse cells (such as human umbilical vein endothelial cells (HUVECs), fibroblasts, or pericytes), growth factors (NO, VEGF, PDGF, and IKVAV), and biomaterial scaffolds (biogenic conduits, soft lithography, micropatterning, and photolithography) in tissue engineering offers a unique strategy for vascularizing long-gap nerve repair [131]. There is a lack of data on combining bioactive factors for improving nerve regrowth and vascularization [132]. Inspiration from analogous tissues is recommended to attain vascularized nerve tissue. In tissue engineering, the various molecules and factors and broad complex pathways are observed to have comprehensive utilization and an impact on the process of angiogenesis [6]. Immobilizing angiogenesis-related biofactors on scaffolds can potentially enhance endothelial cell tube formation, angiogenesis, and revascularization. Dual-biofunctionalized chitosan/collagen scaffolds with IKVAV/VEGF-enhanced neural processes such as neovascularization, axonal regeneration, myelination, and neuromuscular transmission. The designed scaffolds support the proliferation and differentiation of Schwann cells and endothelial cells, aiming to stimulate neovascularization and nerve regeneration. VEGF-related factors from SCs induce arterial differentiation and promote vascular patterns. Endothelial cells secrete nitric oxide to suppress Schwann cell dedifferentiation [132]. These pathways show the complex interaction between vessels and nerves. Controlled delivery of VEGF and rNGF on the surface and core of emulsion electrospun poly (l-lactic acid) (PLLA) nanofiber could affect the regeneration of vascularized nerve tissue [133].

Despite its short half-life, nitric oxide (NO) is a vital signaling molecule in capillary development, vascular growth, and axon regeneration. Three nitric oxide synthase (NOS) isoforms are present in the peripheral nerves. These isoforms include the inducible isoform (iNOS) in various cell types such as macrophages and glial cells. The endothelial isoform (eNOS) is present in the vascular endothelium. The third isoform is the neuronal isoform (nNOS) located in specific neuronal populations [6, 94, 134, 135].

## 7. Effect of Scaffold on Immune Response and Inflammation

PNI causes Wallerian degeneration, which leads to a complex and dynamic microenvironment. The cellular and molecular changes in the microenvironment lead to an imbalance in immune cells and their modulatory factors. The neuroinflammatory response to PNI causes tissue damage, repair processes, inflammation, and rejection postimplantation. The regeneration and inflammation processes consist of various stages and specific populations of cells, including SCs, resident and hematogenous macrophages, and T cells. The immune system, especially T cells, plays a crucial role in nerve regeneration. T cells exhibit both neuroprotective and neurodegenerative roles. In particular, Th2 and Treg cells secrete IL-4 and IL-10, which promote nerve repair. In contrast, Th1 and Th17 cells release IFN-*γ* or IL-17 for enhancing acute inflammatory responses. Modulating the immune response using various therapies that regulate T cells, such as biomaterials design, cell therapy, immunomodulators/cytokines/chemokines, or immunosuppressive drug delivery, is seen as a promising method to improve outcomes related to neuroinflammation and neurodegeneration.

Immunosuppressive therapy is a treatment option for severe traumatic injuries. However, it can be hindered by inflammation that affects multiple tissues. Biomaterial strategies should be considered to address graft rejection and inflammation in various tissues to improve future scaffold designs. The ideal scaffold should have an immunomodulation design that complements nerve regeneration promotion strategies. Material characteristics, such as chemistry, macroarchitecture, surface topography, surface coating, and stiffness, can influence the control of the immune response. Systemic anti-inflammatory or immunomodulation drugs may also enhance nerve regeneration [136–139].

## 8. Clinical Step: Advanced Surgical Technologies and System

Managing peripheral nerve injuries poses significant challenges due to their complex nature and vital functions. Surgeons employ a variety of systems and technologies, ranging from traditional surgical techniques to advanced regenerative medicine, to enhance nerve repair and functional recovery.  Table 6 presents a compilation of several commercial products currently available. This section examines several critical methods and tools currently utilized in this field. Since complete explanations of nerve grafting, regenerative medicine, and tissue engineering were previously provided, this section skips those cases.

### 8.1. Microsurgical Techniques

Microsurgical techniques are paramount in peripheral nerve repair, allowing surgeons to perform exact operations on delicate nerve tissues. The essential elements of microsurgery include [140] the following:

- Operating microscope
- This tool provides the necessary magnification for delicate nerve repair, enhancing visibility and precision.
- Microinstruments
- These specialized tools handle delicate nerve tissues, enabling meticulous manipulation and suturing.
- Sutures and glues
- Ultrafine sutures, as small as 10-0, and biological glues are utilized to reattach nerve ends with minimal trauma to surrounding tissues.

### 8.2. Neurostimulation and Electrostimulation

Neurostimulation techniques are increasingly pivotal in augmenting nerve regeneration and function. Key modalities encompass transcutaneous electrical nerve stimulation (TENS) and implantable nerve stimulators, demonstrating substantial potential in fostering nerve growth and facilitating functional recovery.

#### 8.2.1. Transcutaneous Electrical Nerve Stimulation

TENS is a noninvasive technique that administers electrical impulses to nerves to alleviate pain. It has been widely used to manage various types of chronic pain by targeting sensory fibers to block pain signals. It has been widely used to manage various types of chronic pain by targeting sensory fibers to block pain signals. Research suggests that TENS helps in pain management and enhances neuroplasticity, aiding in the recovery of motor functions. The mechanism involves modulating inhibitory circuits and enhancing reciprocal inhibition, which can be particularly beneficial in conditions such as spasticity following spinal cord injuries [141, 142].

#### 8.2.2. Implantable Nerve Stimulators

Implantable nerve stimulators provide consistent electrical stimulation near the nerve injury site. These devices promote nerve growth and improve functional recovery by directly stimulating the nerves. Studies have shown that using these stimulators can augment the natural regenerative processes of peripheral nerves and contribute to improved reinnervation outcomes. It is important to note that these stimulators are often utilized with other interventions, such as conduits, to guide nerve regrowth effectively [143, 144].

Neurostimulation techniques have the potential to significantly enhance the treatment of nerve injuries by improving synaptic plasticity and promoting axonal regeneration. This innovation promises improved outcomes for individuals with peripheral nerve damage. Further human studies must refine these techniques and facilitate their broader integration into clinical practice.

### 8.3. Robotic Surgical Systems

#### 8.3.1. Robotic Systems

Robotic surgical systems such as da Vinci's have significantly advanced nerve surgeries by elevating precision and overall surgical outcomes. These innovative systems have notably enhanced skill and accuracy in intricate microsurgeries. The da Vinci system, for instance, employs robotic arms to execute delicate operations, enabling meticulous manipulation of nerve tissues. This system offers substantial advantages, including enhanced visualization facilitated by a 3D endoscope, reduced impact of human tremors, and the capability to adjust movements for heightened precision [145, 146].

#### 8.3.2. Laser Devices

Laser devices are increasingly employed in nerve surgeries due to their precision in cutting and sealing nerves, resulting in minimized collateral damage and improved healing outcomes. These devices enable surgeons to perform minimally invasive procedures with increased accuracy, leading to quicker patient recovery and fewer postoperative complications [147].

### 8.4. Exoskeletons and Rehabilitation Devices

#### 8.4.1. Exoskeletons

Wearable robotic devices, or exoskeletons, are essential in postsurgical rehabilitation as they aid patients in movement and muscle-strengthening exercises. These devices play a crucial role in helping patients to regain mobility and promote nerve recovery. For instance, the “Float” upper-limb exoskeleton is specifically designed to enhance motor and functional recovery of the shoulder joint following injuries. It enables patients to perform various activities of daily living (ADLs) by providing support without bearing the weight of the exoskeleton itself [148]. In addition, research has shown that robotic exoskeletons can significantly improve rehabilitation outcomes by offering consistent, controlled assistance and resistance during exercises, which are vital for rebuilding strength and coordination [149].

#### 8.4.2. Virtual Reality (VR) Systems

VR systems are more widely used in rehabilitation to involve patients in interactive exercises stimulating nerve function. Rehabilitation programs based on VR provide an immersive environment that encourages patients to participate in activities that resemble real-life tasks, thus assisting in the recovery of motor skills and cognitive functions. Research has shown that VR systems can improve patient motivation and adherence to rehabilitation protocols, resulting in better functional recovery outcomes [148, 149].

### 8.5. Advanced Prosthetics

Advanced prosthetics offer crucial functional support for cases where nerve injuries result in limb loss, mainly through myoelectric prosthetics and targeted muscle reinnervation (TMR). These advanced prosthetic technologies are crucial for improving amputees' mobility and quality of life, with ongoing innovations to enhance their effectiveness and user experience [150].

#### 8.5.1. Myoelectric Prosthetics

Myoelectric prosthetics are controlled by electrical signals from the remaining muscles, which provide precise and responsive control. These devices use surface electrodes to detect muscle electrical activity, allowing hand opening and closing movements. Despite advancements, challenges persist in achieving natural, intuitive control. The ongoing research is focused on improving the accuracy and usability of these systems.

#### 8.5.2. Targeted Muscle Reinnervation (TMR)

The surgical procedure known as TMR enhances the control of prosthetic limbs by reassigning nerves to different muscle groups. This innovative technique involves transferring residual arm nerves to nearby muscles, which are then reinnervated to provide more natural control signals for the prosthetic. TMR not only improves the functionality of myoelectric prosthetics but also addresses issues such as phantom limb pain and neuromas, thus offering a comprehensive solution for individuals with limb amputations.

## 9. Conclusions

Despite the considerable advancements in medicine, the regeneration process of peripheral neural tissue still presents a challenge. Overcoming gaps more prominent than 30 millimeters is a critical barrier in the regeneration process. Nerve system modeling is challenging due to the diverse cellular structures and complexity of regulating cell-to-cell connections and interactions. Another hurdle is the proper utilization of topographical cues and the timely and spatial release of biochemical signals, which play an active role in regulating the rate of nerve regeneration progression. The complicated and multifaceted nature of the body's response to scaffolds is another confusing factor, ultimately transforming the microenvironment.

The first measure in developing alternative interventions requires a broad understanding of the anatomy and physiology of the peripheral nervous tissue. By understanding the critical elements that lead to adverse functional outcomes following nerve restoration, we can identify opportunities for therapeutic intervention.

One of the critical challenges in the engineering of peripheral nerves pertains to the organization of various sensory and motor neurons within a nerve branch. This process involves simulating a complex mixture of biomaterials next to the need for distinct concentrations of growth factors. Consequently, achieving a universally applicable and fully operational neuron may be an unattainable objective. These elements make the construction process challenging and increase complexity in future assessments [151].

Combining biomaterials and fabrication techniques with cellular technology and tissue engineering is a successful approach to achieving an optimal scaffold that can enhance response rates and expedite regeneration. This multifaceted methodology is currently being utilized to advance the field of scaffold fabrication. The supplementation of biomolecules, transplantation of stem cells, and modulation of cell surfaces through neural scaffold engineering may yield further synergistic effects, potentially exceeding the efficacy of the current clinical method. The other concern is designing an ideal delivery system that effectively controls the spread of the vital cue or drug throughout the entire nervous system regeneration process until functional reinnervation. The interaction between scaffolds and host cells/tissues is a significant factor in nerve regeneration. It is worth noting that cells and neurotrophic factors enrich the scaffold, providing a conducive ECM and microenvironment for the successful regeneration of nerves. Despite extensive research, the optimal cell dose and fate remain ambiguous.

## 10. Future Directions

The current tissue engineering strategies, although helpful, are not perfect. Therefore, there is a need for new techniques that can help improve the regeneration and overall function of engineered neural tissues. In the future, it may be possible to achieve the physiological behavior and better functionality of engineered neural tissues by developing a better understanding of biomaterials and engineering methods and making advancements in cell technology and molecular gradients. The main goal is to increase the number of axons that can regenerate and move through the repair site while also speeding up their growth towards the distal stump.

The construction of engineered neural tissues requires complex interactions multifaced between physicochemical and biological cues, and it is essential that all structures, whether synthetic or natural, are matched in their structural, biological, mechanical, and biochemical compatibility with autografts. Thus, in the future, there is a requirement to develop materials that offer more advantages for reinnervation and more advanced methods for constructing guidance structures.

### 10.1. Data Selection

Relevant article selection was based on specific inclusion criteria. A thorough literature search (up to May 2023) was conducted using different online databases (PubMed, Google Scholar, and Web of Science).

Isolation criteria were based on terms such as “peripheral nerve regeneration,” “stem cells,” “scaffold,” “biomaterials,” and “tissue engineering” in different combinations. Experiment design, sample size, biomaterial selection, fabrication method, seeded cells or growth factors, application, and results were recorded for each study. Original research articles, selected review reports about the objectives, and articles published in English were included. We isolated 151 studies about advances in peripheral nerve tissue engineering.

## Figures and Tables

**Table 1:** Comprehensive summary of the multiple applications of stem cells in neuroregeneration.

Stem cell therapy		Ref
Source	Embryonic stem cells/bone marrow stromal cells/adipose-derived mesenchymal stem cells umbilical cord mesenchymal stem cells/fetal/adult/iPS/fibroblast immunomodulatory cell	[22]
Origin	Autograft/allograft/xenograft	
Administration routes	Intravascular/intrathecal/scaffold loaded/subcuticular/intramuscular/direct injection	[23–26]
Therapeutic actions	Angiogenesis/anti-inflammatory/antiapoptotic/antifibrosis/antioxidation/immunomodulatory/homing/renewal/differentiation/myelination/signaling/cytokine/exosome/neurotrophic factor release	[22, 27, 28]

**Table 2:** Several current studies involve the application of regenerative medicine to peripheral nerve regeneration.

Cells	Material	Fabrication	GF/nano	Animal	Defect	Time of study	Result	Ref
—	PCL	Electrospinning	Conductive GDY	Rat	10-mm sciatic nerve defect model	3 (m)	GDY/PCL NGCs significantly promoted myelination and axonal growth	[83]
SKP-derived Schwann cells (SKP-SCs) or nerve-derived SCs	—	—	—	Rat	12 mm gap in the sciatic nerve	8 (w)	Survival of both cell types' early regeneration in SKP-seeded grafts	[84]
Hair follicle pluripotent stem cells	—	—	—	ICR nude (nu/nu) mice	Impinged sciatic nerve	—	Differentiated into glial fibrillary acidic protein-positive Schwann cells and promoted the recovery of pre-existing axons	[30]
The upper part of the hair follicle containing the hfPSA	—	—	—	Mice	Severed sciatic nerve	—	Implanted hfPS cells grew and promoted the joining of the severed nerve	[31]
Human hair follicle pluripotent stem (hfPS) cells	—	—	—	Mouse	Severed sciatic nerve	—	They differentiated into glial fibrillary-acidic-protein (GFAP)-positive Schwann cells and promoted the recovery of pre-existing axons, leading to nerve generation	[32]
Schwann cell (SC), olfactory ensheathing cell (OEC), or mixed SC/OEC	Poly (dl-lactide-co-glycolide) acid	Conduit	—	Rat	Sciatic nerve transection	—	OEC synergistically improves SC-mediated sciatic nerve repair	[85]
Rat Schwann cells (RSCs)	Porous PVDF/PCL composite	Cast/annealing-solvent displacement method	—	Rat	Sciatic nerve model for 4 months	4 (m)	Piezoelectric scaffold	[86]
—	Collagen/PLA	Electrospun	—	—	—	—	—	[87]
Schwann cells	PCL and CNTs	Aligned electrospinning	BDNF	Rat	10 mm sciatic nerve defect model	—	Promote sciatic nerve regeneration and functional recovery	[88]
	Pure porcine decellularized nerve matrix/proanthocyanidins	Electrospinning	—	Rat	8 mm sciatic nerve	12 (w)	Excellent ability to transmit electrical signals	[82]
—	Soft carbon nanotubes@gelatin methacryloyl/poly (L-lactic acid) (CNTs@GelMA/PLLA)	—	—	Rat	10-mm sciatic nerve defect	12 (w)	—	[89]
—	PVDF/poly PLCL film SF/PEDOT cryogel	Gradient freezing method In situ polymerization	—	Rat	Sciatic nerve defect	12 (w)	—	[90]
—	(rGO with GelM) and (PCL) nerve conduits	Electrostatic spinning	BMSC-derived extracellular vesicles	Rat	10 mm sciatic defect	12 (w)	Promote peripheral nerve regeneration and neurological function recovery	[91]
—	Hyaluronic acid methacrylate	Photocrosslinked hydrogel	MSC-Exos	Rat	SNCI model	1 and 2 (w)	Inhibit the infiltration of macrophages and the expression of the proinflammatory factors IL-1*β* and TNF*α*	[92]
—	Pyrrole electroconductive hydrogels	—	BMSC-Exos	Rat	Diabetic sciatic nerve crush injury	4 (w)	Enhance myelinated axonal regrowth	[81]
—	Chitin conduits + injectable decellularized matrix hydrogel	Physical, chemical, and enzymatic digestions	ADSC	Rat	Sciatic nerve defects	—	Improved peripheral nerve regeneration	[93]

**Table 3:** Alternative therapy for nerve defects.

Nerve graft/replacement	Inert	Immune rejection	Topographical cues
Autograft	+	−	+
Allograft	+	+	+
Processed acellular nerve allografts	+	−	+
Hollow tube synthetic conduits	+	−	−

**Table 4:** FDA-approved nerve product (conduit, cuff, or wrap).

Biomaterial	Product^∗^
Collagen type I	NeuraGen™ (K011168, 2001)
	NeuroMatrix™ (K012814, 2001)
	NeuraWrap™ (K041620, 2004)
	NeuraFlex™ (K012814, 2001)
	NeuroMend™ (K060952, 2006)
	Nerve cuff (K132660)
	Flexible collagen nerve cuff (K131541)
	NeuroCap™ (K152648, 2016)

Type I collagen and glycosaminoglycan (chondroitin-6-sulfate)	NeuraGen™ 3D (K130557, 2014)
	NeuraGen®3D nerve guide matrix (K163457)

Type I acellular porcine collagen membrane	Cova™ ORTHO-NERVE (K103081, 2012)

Collagen matrix derived from porcine pericardium	NervAlign® nerve cuff (K202234)

Collagen type I + absorbable polymeric suture filament	Reinforced flexible collagen nerve cuff (K170656, 2017)

Polyglycolic acid (PGA)	Neurotube™ (K983007, 1999)
	NeuroFlex™ (K131541, 2014)
	Nerbridge™ (K152967, 2016)

Polyglycolic acid (PGA) and type I and III collagen and porcine	Nerbridge™ (K152967)

Poly D, L-lactide-co-e-caprolactone (PLCL)	Neurolac™ (K032115, 2003; K050573, 2005; K112267, 2011) (K112267)
	Nerve capping device (K152684)

Polyvinyl alcohol (PVA)	Salu tunnel™ (2010, K100382)
	Salubridge (K002098, 2001)

Porcine small intestine submucosa	AxoGaurd™ (K031069, 2003) (K162741)
	Axogen (K163446)

Porcine small intestinal submucosa; primarily types I, III, IV, and VI collagen	Axoguard nerve connector (K162741)

Human nerve allograft	Avance

Chitosan	Reaxon™ Plus (K143711, 2015) (K180222)
	NeuroShield (K190246)

Hyaluronic acid and alginate	VersaWrap nerve protecto (K201631)

^∗^The products mentioned have been sourced from the FDA website.

**Table 5:** The table overviews various scaffold morphologies' current advantages, disadvantages, and prospective strategies.

Scaffold morphologies	Fabrication	Advantages	Disadvantage	Prospective	Ref
Porous	(i) Templating (ii) Freeze-drying (iii) Electrospinning (iv) 3D bioprinting	(i) Infiltration	(i) Fibrous scarring (ii) Obstructing cellular communication	(i) Optimizing the size of pores	[71]

Hydrogels	(i) Physical (ii) Chemical (iii) Hybrid bonding	(i) 3D hydration networks (ii) Support of cell adhesion, infiltration, proliferation, migration, and differentiation (iii) Excellent biological activity, biodegradability, and biocompatibility (iv) Local drug delivery carriers (v) Fillers for nerve-guiding conduits (vi) Appropriated mechanical properties	(i) Poor prognosis in severe long-gap nerve defect (ii) Difficult to sterilize and handle (iii) Fragile nature (iv) Low stability (v) low adhesion	(i) Enhance the microstructure, characteristics, and mechanical properties of hydrogels (ii) Responsiveness of the hydrogels as delivery vehicles	[106, 107]

Fibrous	(i) Electrospinning (ii) Templating (iii) Phase separation self-assembly	(i) Resemblance to native extracellular matrix (ii) Support cell adhesion, outgrowth, and differentiation (iii) High surface area to volume ratio (iv) High mechanical strength (v) Good suturing and grafting	(i) Regulate fiber diameter and alignment	(i) Mechanical properties (ii) Integration (iii) Multifunctionality	[72]

**Table 6:** List of various commercial products that are currently available.

Model	Product name and company	Material	Description
Nerve conduits (nerve guides)	Axoguard nerve connector AxoGen, Inc	Porcine small intestine submucosa (SIS)	(i) Connecting severed nerve ends with gaps up to 5 mm (ii) Providing a protected environment for nerve regeneration
	NeuraGen nerve guide Integra LifeSciences	Type I collagen	(i) A biodegradable scaffold that supports nerve regrowth
	NeuroTube Synovis Micro Companies Alliance	Polyglycolic acid (PGA)	(i) Absorbed by the body after the nerve has regenerated (ii) Commonly used in microsurgical procedures to repair small nerves
	Stryker NeuroMend collagen nerve wrap Stryker	Type I collagen	(i) Protecting and supporting regenerating nerves (ii) Helping to reduce scar tissue formation (iii) Beneficial in complex nerve repairs where direct suturing is not feasible

Nerve Allografts	Avance nerve graft AxoGen, Inc	Decellularized human nerve allograft	(i) Retaining the extracellular matrix components necessary to support nerve regeneration (ii) Minimizing immune response (iii) Reducing the incidence of persistent nerve pain compared to other methods (iv) Supplied sterile with three years shelf life (kept frozen at or below −40° C/F)
	Reprocessed human nerve allografts	Cadaveric nerve	(i) Using techniques such as the administration of concurrent immunosuppressants (ii) Pretreating with radiation and lyophilization to combat the problem of immunogenicity (iii) Off-the-shelf convenience (iv) Often sourced from human tissue banks

Nerve wraps	Axoguard nerve protector AxoGen, Inc	Porcine small intestine submucosa (SIS)	(i) Providing a physical barrier to protect injured nerves from soft tissue attachments during the healing process (ii) Promoting vascularization and remodeling into the patient's tissue (iii) Facilitating nerve regeneration while preventing nerve entrapment (iv) Minimizing the potential for soft tissue attachments
	NeuraWrap Integra LifeSciences	Type I collagen	(i) Minimizing scar tissue formation and supporting nerve healing (ii) Biodegradable (iii) Supporting the natural regeneration process of the nerve

Bioengineered nerve scaffolds	Reaxon nerve guide (Medovent GmbH)	Chitosan	(i) A synthetic nerve guide supports nerve regeneration (ii) Gradually degrades as the nerve heals (iii) Easy to suture
	NeuroRegen Scaffold (MedXpress)	Collagen	(i) Combining a physical scaffold with growth factors to enhance nerve repair
	Neurotube (Synovis Micro Companies Alliance)	Polyglycolic acid (PGA)	(i) Designing to support nerve regeneration (ii) Being absorbed by the body over time (iii) Efficacy in bridging nerve gaps and supporting axonal growth
	Neurolac nerve guide (Polyganics)	Poly (DL-lactide-*ε*-caprolactone)	(i) Temporary scaffold for nerve regeneration (ii) Degrading naturally over time, eliminating the need for surgical removal (iii) Reducing tension and enhanced nerve regeneration

Electrical stimulation devices (neurostimulation devices)	ActiGait (Neurodan)	—	(i) Providing electrical stimulation to nerves (ii) Aiding in nerve regeneration
	ReActiv8x (Mainstay Medical)	—	(i) An implantable neurostimulation system (ii) Activating and conditioning the muscles that stabilize the lower back (iii) Supporting nerve health indirectly

## Data Availability

The data supporting this review are from previously reported studies and datasets, which have been cited.

## References

[1] Zhuang P., Sun A. X., An J., Chua C. K., Chew S. Y. (2018). 3D neural tissue models: from spheroids to bioprinting. *Biomaterials*.

[2] Sensharma P., Madhumathi G., Jayant R. D., Jaiswal A. K. (2017). Biomaterials and cells for neural tissue engineering: current choices. *Electronics*.

[3] Tuffaha S., Sarhane K., Qiu C., Harris T., Hanwright P., Mao H. Q. (2023). Translational bioengineering strategies for peripheral nerve regeneration: opportunities, challenges, and novel concepts. *Neural Regeneration Research*.

[4] Liu C. Y., Yin G., Sun Y. D. (2020). Effect of exosomes from adipose‐derived stem cells on the apoptosis of Schwann cells in peripheral nerve injury. *CNS Neuroscience and Therapeutics*.

[5] Du J., Chen H., Qing L., Yang X., Jia X. J. B. s. (2018). Biomimetic neural scaffolds: a crucial step towards optimal peripheral nerve regeneration. *Biomaterials Science*.

[6] Gu X., Ding F., Williams D. F. (2014). Neural tissue engineering options for peripheral nerve regeneration. *Electronics*.

[7] Barros C. S., Franco S. J., Müller U. J. C. S. H. p.i.b. (2011). Extracellular matrix: functions in the nervous system. *Cold Spring Harbor Perspectives in Biology*.

[8] Dingal P. C. D. P., Discher D. E. (2014). Combining insoluble and soluble factors to steer stem cell fate. *Nature Materials*.

[9] Qian Y., Lin H., Yan Z., Shi J., Fan C. J. M. T. (2021). Functional nanomaterials in peripheral nerve regeneration: scaffold design, chemical principles and microenvironmental remodeling. *Materials Today*.

[10] Kapur T. A., Shoichet M. S. (2004). Immobilized concentration gradients of nerve growth factor guide neurite outgrowth. *Journal of Biomedical Materials Research Part A*.

[11] Min Q., Parkinson D. B., Dun X.-P. (2021). Migrating Schwann cells direct axon regeneration within the peripheral nerve bridge. *GLIA*.

[12] Fairbairn N. G., Meppelink A. M., Ng-Glazier J., Randolph M. A., Winograd J. M. (2015). Augmenting peripheral nerve regeneration using stem cells: a review of current opinion. *World Journal of Stem Cells*.

[13] Lu P., Wang G., Qian T. (2021). The balanced microenvironment regulated by the degradants of appropriate PLGA scaffolds and chitosan conduit promotes peripheral nerve regeneration. *Materials Today Bio*.

[14] Marinval N. A.-O. X., Chew S. A.-O. Mechanotransduction assays for neural regeneration strategies: a focus on glial cells. *Electronics*.

[15] Gnavi S., Barwig C., Freier T., Haastert-Talini K., Grothe C., Geuna S. J. I. r.o.n. (2013). The use of chitosan-based scaffolds to enhance regeneration in the nervous system. *International Review of Neurobiology*.

[16] Endo T., Kadoya K., Suzuki T. (2022). Mature but not developing Schwann cells promote axon regeneration after peripheral nerve injury. *Npj Regenerative Medicine*.

[17] Cui L., Jiang J., Wei L. (2008). Transplantation of embryonic stem cells improves nerve repair and functional recovery after severe sciatic nerve axotomy in rats. *Stem Cells*.

[18] Sivanarayanan T., Bhat I. A., Sharun K. (2023). Allogenic bone marrow-derived mesenchymal stem cells and its conditioned media for repairing acute and sub-acute peripheral nerve injuries in a rabbit model. *Tissue and Cell*.

[19] Yu P., Zhang G., Hou B. (2023). Effects of ECM proteins (laminin, fibronectin, and type IV collagen) on the biological behavior of Schwann cells and their roles in the process of remyelination after peripheral nerve injury. *Frontiers in Bioengineering and Biotechnology*.

[20] Yılmaz M. M., Akdere Ö. E., Gümüşderelioğlu M. (2023). Biological nerve conduit model with de-epithelialized human amniotic membrane and adipose-derived mesenchymal stem cell sheet for repair of peripheral nerve defects. *Cell and Tissue Research*.

[21] Wang Y., Guo Z. y., Sun X., Xu X. l., Peng J., Wang Y. J. N. r.r. (2015). Human umbilical cord mesenchymal stem cells promote peripheral nerve repair via paracrine mechanisms. *Neural Regeneration Research*.

[22] Bonaventura G., Munafò A., Bellanca C. M. (2021). Stem cells: innovative therapeutic options for neurodegenerative diseases?. *Cells*.

[23] Hachimi-Idrissi S. (2023). Stem cell therapy in neurological disorders: promises and concerns. *Exploration of Neuroprotective Therapy*.

[24] Mirahmadi M., Rezanejadbardaji H., Irfan-Maqsood M., Mokhtari M., Naderi-Meshkin H. (2016). Stem cell therapy for neurodegenerative diseases: strategies for regeneration against degeneration. *Cell Therapy and Regenerative Medicine Journal*.

[25] Lavorato A., Raimondo S., Boido M. (2021). Mesenchymal stem cell treatment perspectives in peripheral nerve regeneration: systematic review. *International Journal of Molecular Sciences*.

[26] Liu B., Kong Y., Shi W. (2022). Exosomes derived from differentiated human ADMSC with the Schwann cell phenotype modulate peripheral nerve-related cellular functions. *Bioactive Materials*.

[27] Rao Z., Lin Z., Song P., Quan D., Bai Y. (2022). Biomaterial-based schwann cell transplantation and schwann cell-derived biomaterials for nerve regeneration. *Frontiers in Cellular Neuroscience*.

[28] Yin Q., Zou T., Sun S., Yang D. (2023). Cell therapy for neuropathic pain. *Frontiers in Molecular Neuroscience*.

[29] Zack-Williams S. D., Butler P. E., Kalaskar D. M. (1948). Current progress in use of adipose derived stem cells in peripheral nerve regeneration. *World Journal of Stem Cells*.

[30] Amoh Y., Aki R., Hamada Y. (2012). Nestin-positive hair follicle pluripotent stem cells can promote regeneration of impinged peripheral nerve injury. *The Journal of Dermatology*.

[31] Amoh Y., Hamada Y., Aki R., Kawahara K., Hoffman R. M., Katsuoka K. (2010). Direct transplantation of uncultured hair-follicle pluripotent stem (hfPS) cells promotes the recovery of peripheral nerve injury. *Journal of Cellular Biochemistry*.

[32] Amoh Y., Kanoh M., Niiyama S. (2009). Human hair follicle pluripotent stem (hfPS) cells promote regeneration of peripheral‐nerve injury: an advantageous alternative to ES and iPS cells. *Journal of Cellular Biochemistry*.

[33] Spyridopoulos T., Lambropoulou M., Pagonopoulou O. (2015). Regenerated nerve defects with a nerve conduit containing dental pulp stem cells in pigs: an immunohistochemical and electrophysiological evaluation. *Electronics*.

[34] Zhou X., Yu M., Chen D. (2023). Chitosan nerve grafts incorporated with SKP-SC-EVs induce peripheral nerve regeneration. *Tissue Engineering and Regenerative Medicine*.

[35] Ikeda M., Uemura T., Takamatsu K. (2014). Acceleration of peripheral nerve regeneration using nerve conduits in combination with induced pluripotent stem cell technology and a basic fibroblast growth factor drug delivery system. *Journal of Biomedical Materials Research Part A*.

[36] Uemura T., Takamatsu K., Ikeda M. (2011). A tissue-engineered bioabsorbable nerve conduit created by three-dimensional culture of induced pluripotent stem cell-derived neurospheres. *Bio-Medical Materials and Engineering*.

[37] Behbehani M., Glen A., Taylor C. S., Schuhmacher A., Claeyssens F., Haycock J. W. (2018). Pre-clinical evaluation of advanced nerve guide conduits using a novel 3D in vitro testing model. *Electronics*.

[38] Yurie H., Ikeguchi R., Aoyama T. (2017). The efficacy of a scaffold-free Bio 3D conduit developed from human fibroblasts on peripheral nerve regeneration in a rat sciatic nerve model. *PLoS One*.

[39] Su Q., Nasser M. I., He J. (2022). Engineered Schwann cell-based therapies for injury peripheral nerve reconstruction. *Frontiers in Cellular Neuroscience*.

[40] Tang X., Xue C Fau - Wang Y., Wang Y Fau - Ding F. (2012). Bridging peripheral nerve defects with a tissue engineered nerve graft composed of an in vitro cultured nerve equivalent and a silk fibroin-based scaffold. *Electronics*.

[41] Shakhbazau A., Kawasoe J., Hoyng S. A. (2012). Early regenerative effects of NGF-transduced Schwann cells in peripheral nerve repair. *Molecular and Cellular Neuroscience*.

[42] Zhang Y.-J., Zhang W., Lin C.-G. (2012). Neurotrophin-3 gene modified mesenchymal stem cells promote remyelination and functional recovery in the demyelinated spinal cord of rats. *Journal of the Neurological Sciences*.

[43] Hsu M.-N., Liao H.-T., Li K.-C. (2017). Adipose-derived stem cell sheets functionalized by hybrid baculovirus for prolonged GDNF expression and improved nerve regeneration. *Biomaterials*.

[44] Luo H., Xu C., Liu Z. (2019). Neural differentiation of bone marrow mesenchymal stem cells with human brain‐derived neurotrophic factor gene‐modified in functionalized self‐assembling peptide hydrogel in vitro. *Journal of Cellular Biochemistry*.

[45] Zhang Y.-r., Huang W. h., Ka K. (2015). Repair of peripheral nerve defects with chemically extracted acellular nerve allografts loaded with neurotrophic factors-transfected bone marrow mesenchymal stem cells. *Neural Regeneration Research*.

[46] Poongodi R., Chen Y.-L., Yang T.-H. (2021). Bio-scaffolds as cell or exosome carriers for nerve injury repair. *International Journal of Molecular Sciences*.

[47] Marquardt L. M., Sakiyama-Elbert S. E. (2013). Engineering peripheral nerve repair. *Electronics*.

[48] Li X., Guan Y., Li C. (2022). Therapy, Immunomodulatory effects of mesenchymal stem cells in peripheral nerve injury. *Stem Cell Research & Therapy*.

[49] Yi S., Xu L., Gu X. (2019). Scaffolds for peripheral nerve repair and reconstruction. *Experimental Neurology*.

[50] Sulaiman W., Dreesen T., Nguyen D. J. N. (2018). Single local application of TGF-*β* promotes a proregenerative state throughout a chronically injured nerve. *Neurosurgery*.

[51] Tiwari A. A.-O., Lokai T., Albin B., Yang I. A.-O. (2022). A review on the technological advances and future perspectives of axon guidance and regeneration in peripheral nerve repair. *Bioengineering*.

[52] Patel R., Santhosh M., Dash J. K. (2019). Ile-Lys-Val-ala-Val (IKVAV) peptide for neuronal tissue engineering. *Polymers for Advanced Technologies*.

[53] Motta C. M. M., Endres K. J., Wesdemiotis C., Willits R. K., Becker M. L. (2019). Enhancing Schwann cell migration using concentration gradients of laminin-derived peptides. *Biomaterials*.

[54] Rao S. S., Winter J. O. (2009). Adhesion molecule-modified biomaterials for neural tissue engineering. *Frontiers in Neuroengineering*.

[55] Sánchez M., Anitua E., Delgado D. (2017). Platelet-rich plasma, a source of autologous growth factors and biomimetic scaffold for peripheral nerve regeneration. *Expert Opinion on Biological Therapy*.

[56] Ortiz A. d. C., Fideles S. O. M., Pomini K. T. (2022). Potential of fibrin glue and mesenchymal stem cells (MSCs) to regenerate nerve injuries: a systematic review. *Cells*.

[57] Siemionow M., Bozkurt M Fau - Zor F., Zor F. Regeneration and repair of peripheral nerves with different biomaterials: review. *Electronics*.

[58] Wilson E. R., Della-Flora Nunes G. A.-O., Weaver M. R., Frick L. R., Feltri M. L. (2021). Schwann cell interactions during the development of the peripheral nervous system. *Developmental Neurobiology*.

[59] Nieuwenhuis B., Haenzi B., Andrews M. R., Verhaagen J., Fawcett J. W. J. B. R. (2018). Integrins promote axonal regeneration after injury of the nervous system. *Biological Reviews*.

[60] Ryan A. J., Lackington W. A., Hibbitts A. J. (2017). A physicochemically optimized and neuroconductive biphasic nerve guidance conduit for peripheral nerve repair. *Advanced Healthcare Materials*.

[61] Chen X., Ge X., Qian Y. (2020). Electrospinning multilayered scaffolds loaded with melatonin and Fe3O4 magnetic nanoparticles for peripheral nerve regeneration. *Advanced Functional Materials*.

[62] Yamamoto T., Osako Y., Ito M. (2016). Trophic effects of dental pulp stem cells on Schwann cells in peripheral nerve regeneration. *Cell Transplantation*.

[63] Pramanik S., Muthuvijayan V. (2022). Electrospun nanofibrous scaffolds for neural tissue engineering. *Springer*.

[64] Park D., Kim D., Park S. J. (2022). Micropattern-based nerve guidance conduit with hundreds of microchannels and stem cell recruitment for nerve regeneration. *Npj Regenerative Medicine*.

[65] Huang L., Zhu L., Shi X. (2018). A compound scaffold with uniform longitudinally oriented guidance cues and a porous sheath promotes peripheral nerve regeneration in vivo. *Acta Biomater*.

[66] Takeuchi H., Ikeguchi R., Aoyama T. (2020). A scaffold‐free Bio 3D nerve conduit for repair of a 10‐mm peripheral nerve defect in the rats. *Microsurgery*.

[67] Hu X., Huang J., Ye Z. (2009). A novel scaffold with longitudinally oriented microchannels promotes peripheral nerve regeneration. *Tissue Engineering Part A*.

[68] Arslantunali D., Dursun T., Yucel D., Hasirci N., Hasirci V. (2014). Peripheral nerve conduits: technology update. *Medical devices (Auckland, N.Z.)*.

[69] Ellermann E., Meyer N., Cameron R. E., Best S. M. (2023). In vitro angiogenesis in response to biomaterial properties for bone tissue engineering: a review of the state of the art. *Regenerative Biomaterials*.

[70] Muheremu A., Ao Q. (2015). Past, present, and future of nerve conduits in the treatment of peripheral nerve injury. *BioMed Research International*.

[71] Wan T., Wang Y.-L., Zhang F.-S. (2023). The porous structure of peripheral nerve guidance conduits: features, fabrication, and implications for peripheral nerve regeneration. *International Journal of Molecular Sciences*.

[72] Subramanian A., Krishnan Um Fau - Sethuraman S., Sethuraman S. Development of biomaterial scaffold for nerve tissue engineering: biomaterial mediated neural regeneration. *Electronics*.

[73] Oh S. H., Kang J. G., Kim T. H. (2018). Enhanced peripheral nerve regeneration through asymmetrically porous nerve guide conduit with nerve growth factor gradient. *Journal of Biomedical Materials Research Part A*.

[74] Zhang L., Sun R., Wang B., Lang Y., Chang M. W., Biomaterials P. (2023). Polycaprolactone/multi-walled carbon nanotube nerve guidance conduits with tunable channels fabricated via novel extrusion-stretching method for peripheral nerve repair. *International Journal of Polymeric Materials and Polymeric Biomaterials*.

[75] Lizarraga‐Valderrama L. R., Ronchi G., Nigmatullin R. (2021). Preclinical study of peripheral nerve regeneration using nerve guidance conduits based on polyhydroxyalkanaotes. *Bioengineering & Translational Medicine*.

[76] Georgiou M., Golding J. P., Loughlin A. J., Kingham P. J., Phillips J. B. (2015). Engineered neural tissue with aligned, differentiated adipose-derived stem cells promotes peripheral nerve regeneration across a critical sized defect in rat sciatic nerve. *Biomaterials*.

[77] Bikis C., Degrugillier L., Thalmann P. (2018). Three-dimensional imaging and analysis of entire peripheral nerves after repair and reconstruction. *Journal of Neuroscience Methods*.

[78] Gao S., Chen X., Lu B., Meng K., Zhang K.-Q., Zhao H. (2023). Recent advances on nerve guide conduits based on textile methods. *Smart Materials in Medicine*.

[79] Abzan N., Kharaziha M., Labbaf S. J. M. (2019). Design, Development of three-dimensional piezoelectric polyvinylidene fluoride-graphene oxide scaffold by non-solvent induced phase separation method for nerve tissue engineering. *Materials & Design*.

[80] Rao F., Yuan Z., Li M. (2019). Expanded 3D nanofibre sponge scaffolds by gas-foaming technique enhance peripheral nerve regeneration. *Artificial Cells, Nanomedicine, and Biotechnology*.

[81] Yang Q., Su S., Liu S. (2023). Exosomes-loaded electroconductive nerve dressing for nerve regeneration and pain relief against diabetic peripheral nerve injury. *Bioactive Materials*.

[82] Kong Y., Xu J., Han Q. (2022). Electrospinning porcine decellularized nerve matrix scaffold for peripheral nerve regeneration. *International Journal of Biological Macromolecules*.

[83] Li X., He N., Li X. (2023). Graphdiyne-loaded polycaprolactone nanofiber scaffold for peripheral nerve regeneration. *Journal of Colloid and Interface Science*.

[84] Walsh S., Biernaskie J., Kemp S. W. P., Midha R. (2009). Supplementation of acellular nerve grafts with skin derived precursor cells promotes peripheral nerve regeneration. *Neuroscience*.

[85] You H., Wei L., Liu Y. (2011). Olfactory ensheathing cells enhance Schwann cell-mediated anatomical and functional repair after sciatic nerve injury in adult rats. *Experimental Neurology*.

[86] Cheng Y., Xu Y., Qian Y., Chen X., Ouyang Y., Yuan W.-E. (2020). 3D structured self-powered PVDF/PCL scaffolds for peripheral nerve regeneration. *Nano Energy*.

[87] Ma J., Li J., Hu S. (2022). Collagen modified anisotropic PLA scaffold as a base for peripheral nerve regeneration. *Macromolecular Bioscience*.

[88] Pi W., Zhang Y., Li L. (2022). Polydopamine-coated polycaprolactone/carbon nanotube fibrous scaffolds loaded with brain-derived neurotrophic factor for peripheral nerve regeneration. *Biofabrication*.

[89] Yang Y., Yin X., Wang H. (2023). Engineering a wirelessly self-powered and electroconductive scaffold to promote peripheral nerve regeneration. *Nano Energy*.

[90] Ma Y., Wang H., Wang Q., Cao X., Gao H. (2023). Piezoelectric conduit combined with multi-channel conductive scaffold for peripheral nerve regeneration. *Chemical Engineering Journal*.

[91] Zhang W., Fang X. X., Li Q. C., Pi W., Han N. (2023). Reduced graphene oxide-embedded nerve conduits loaded with bone marrow mesenchymal stem cell-derived extracellular vesicles promote peripheral nerve regeneration. *Neural Regeneration Research*.

[92] Liu Z., Tong H., Li J. (2022). Low-stiffness hydrogels promote peripheral nerve regeneration through the rapid release of exosomes. *Frontiers in Bioengineering and Biotechnology*.

[93] Li Y., Chen Z., Zhou J. (2023). Combining chitin biological conduits with injectable adipose tissue-derived decellularised matrix hydrogels loaded with adipose-derived mesenchymal stem cells for the repair of peripheral nerve defects in rats. *Colloids and Surfaces A: Physicochemical and Engineering Aspects*.

[94] Zhu J., Wang Y., Zhong L., Pan F., Wang J. J. D. D. (2021). Advances in tissue engineering of vasculature through three‐dimensional bioprinting. *Developmental Dynamics*.

[95] Adutler-Lieber S., Zaretsky I., Platzman I. (2014). Engineering of synthetic cellular microenvironments: implications for immunity. *Journal of Autoimmunity*.

[96] Pooshidani Y., Zoghi N., Rajabi M., Haghbin Nazarpak M., Hassannejad Z. J. J. o.M. S. M. i.M. (2021). Fabrication and evaluation of porous and conductive nanofibrous scaffolds for nerve tissue engineering. *Journal of Materials Science: Materials in Medicine*.

[97] Zhao Y., Wang Y., Gong J. (2017). Chitosan degradation products facilitate peripheral nerve regeneration by improving macrophage-constructed microenvironments. *Biomaterials*.

[98] Wood M. D., Willits R. K. J. (2009). Applied electric field enhances DRG neurite growth: influence of stimulation media, surface coating and growth supplements. *Journal of Neural Engineering*.

[99] Moskow J., Ferrigno B., Mistry N. (2019). Review: bioengineering approach for the repair and regeneration of peripheral nerve. *Bioactive Materials*.

[100] Escobar A., Serafin A., Carvalho M. R. (2023). Electroconductive poly(3,4-ethylenedioxythiophene) (PEDOT) nanoparticle-loaded silk fibroin biocomposite conduits for peripheral nerve regeneration. *Advanced Composites and Hybrid Materials*.

[101] Rahman M., Mahady Dip T., Padhye R., Houshyar S. (2023). Review on electrically conductive smart nerve guide conduit for peripheral nerve regeneration. *Journal of Biomedical Materials Research Part A*.

[102] Zeng Z., Yang Y., Deng J., Saif Ur Rahman M., Sun C., Xu S. (2022). Physical stimulation combined with biomaterials promotes peripheral nerve injury repair. *Bioengineering*.

[103] Maeng W.-Y., Tseng W.-L., Li S., Koo J., Hsueh Y.-Y. (2022). Electroceuticals for peripheral nerve regeneration. *Biofabrication*.

[104] Doblado L. R., Martínez-Ramos C., Pradas M. M. (2021). Biomaterials for neural tissue engineering. *Frontiers in Nanotechnology*.

[105] Braga Silva J., Marchese G. M., Cauduro C. G., Debiasi M. (2017). Nerve conduits for treating peripheral nerve injuries: a systematic literature review. *Hand Surgery and Rehabilitation*.

[106] Zhang M., Li L., An H., Zhang P., Liu P. (2021). Repair of peripheral nerve injury using hydrogels based on self-assembled peptides. *Gels*.

[107] Liu Y., Zhang X., Xiao C., Liu B. (2023). Engineered hydrogels for peripheral nerve repair. *Materials Today Bio*.

[108] Kong Y., Shi W., Zhang D. (2021). Injectable, antioxidative, and neurotrophic factor-deliverable hydrogel for peripheral nerve regeneration and neuropathic pain relief. *Applied Materials Today*.

[109] Huang L., Yang X., Deng L. (2021). Biocompatible chitin hydrogel incorporated with PEDOT nanoparticles for peripheral nerve repair. *ACS Applied Materials & Interfaces*.

[110] Liu Y., Zhang X., Xiao C., Liu B. J. M. T. B. (2023). Engineered hydrogels for peripheral nerve repair. *Materials Today Bio*.

[111] McGrath A. M., Novikova L. N., Novikov L. N., Wiberg M. J. B. r.b. (2010). BD™ PuraMatrix™ peptide hydrogel seeded with Schwann cells for peripheral nerve regeneration. *Brain Research Bulletin*.

[112] Cai Y., Huang Q., Wang P. (2022). Conductive hydrogel conduits with growth factor gradients for peripheral nerve repair in diabetics with non‐suture tape. *Advanced Healthcare Materials*.

[113] Isaacs J., Browne T. J. H. (2014). Overcoming short gaps in peripheral nerve repair: conduits and human acellular nerve allograft. *Hand*.

[114] Al-Hadeethi Y., Nagarajan A., Hanuman S. (2023). Schwann cell-matrix coated PCL-MWCNT multifunctional nanofibrous scaffolds for neural regeneration. *RSC Advances*.

[115] Hu X., Xu Y., Xu Y., Li Y., Guo J. (2023). Nanotechnology and nanomaterials in peripheral nerve repair and reconstruction. *Micro/Nano Technologies*.

[116] Su Y., Toftdal M. S., Le Friec A., Dong M., Han X., Chen M. (2021). 3D electrospun synthetic extracellular matrix for tissue regeneration. *Small Science*.

[117] Sharifi M., Farahani M. K., Salehi M. (2022). Engineering, exploring the physicochemical, electroactive, and biodelivery properties of metal nanoparticles on peripheral nerve regeneration.

[118] Amini N., Milan P. B., Sarmadi V. H. (2022). Microorganism-derived biological macromolecules for tissue engineering. *Electronics*.

[119] Kvist M., Sondell M., Kanje M., Dahlin L. B. J. J. o.p.s., surgery h. (2011). Regeneration in, and properties of, extracted peripheral nerve allografts and xenografts. *Journal of Plastic Surgery and Hand Surgery*.

[120] Moore A. M., MacEwan M., Santosa K. B. (2011). Acellular nerve allografts in peripheral nerve regeneration: a comparative study. *Muscle & Nerve*.

[121] Amini N., Hivechi A., Asadpour S. (2023). Fabrication and characterization of bilayer scaffolds made of decellularized dermis/nanofibrous collagen for healing of full-thickness wounds. *Electronics*.

[122] Zaminy A., Sayad-Fathi S., Kasmaie F. M., Jahromi Z., Zendedel A. J. N. R. R. (2021). Decellularized peripheral nerve grafts by a modified protocol for repair of rat sciatic nerve injury. *Neural Regeneration Research*.

[123] Pedroza-Montoya F.-E., Tamez-Mata Y.-A., Simental-Mendía M. (2022). Repair of ovine peripheral nerve injuries with xenogeneic human acellular sciatic nerves prerecellularized with allogeneic Schwann-like cells—an innovative and promising approach. *Regenerative Therapy*.

[124] Lu L.-J., Sun J.-B., Liu Z.-G., Gong X., Cui J.-L., Sun X.-G. (2009). Immune responses following mouse peripheral nerve xenotransplantation in rats. *Journal of Biomedicine and Biotechnology*.

[125] Boyer R. B., Sexton K. W., Rodriguez-Feo C. L. (2015). Adjuvant neurotrophic factors in peripheral nerve repair with chondroitin sulfate proteoglycan-reduced acellular nerve allografts. *Journal of Surgical Research*.

[126] Zilic L., Wilshaw S. P., Haycock J. W. J. B. (2016). Decellularisation and histological characterisation of porcine peripheral nerves. *Biotechnology and Bioengineering*.

[127] Yu H., Peng J., Guo Q. (2009). Improvement of peripheral nerve regeneration in acellular nerve grafts with local release of nerve growth factor. *Microsurgery*.

[128] Yu T., Ao Q., Ao T. (2022). Preparation and assessment of an optimized multichannel acellular nerve allograft for peripheral nerve regeneration, n/a(n/a). *Acta Biomaterialia*.

[129] Hsu M.-W., Chen S.-H., Tseng W.-L. (2023). Physical processing for decellularized nerve xenograft in peripheral nerve regeneration. *Frontiers in Bioengineering and Biotechnology*.

[130] Gulati A. K. (1988). Evaluation of acellular and cellular nerve grafts in repair of rat peripheral nerve. *Journal of Neurosurgery*.

[131] Muangsanit P., Shipley R. J., Phillips J. A.-O. (1932). Vascularization strategies for peripheral nerve tissue engineering. *Electronics*.

[132] Li G. A.-O., Han Q., Lu P. Construction of dual-biofunctionalized chitosan/collagen scaffolds for simultaneous neovascularization and nerve regeneration. *Electronics*.

[133] Xia B., Lv Y. Dual-delivery of VEGF and NGF by emulsion electrospun nanofibrous scaffold for peripheral nerve regeneration. *Electronics*.

[134] Lee Y. B., Polio S Fau - Lee W., Lee W Fau - Dai G. (2010). Bio-printing of collagen and VEGF-releasing fibrin gel scaffolds for neural stem cell culture. *Electronics*.

[135] Sondell M., Lundborg G Fau - Kanje M., Kanje M. (1999). Vascular endothelial growth factor has neurotrophic activity and stimulates axonal outgrowth, enhancing cell survival and Schwann cell proliferation in the peripheral nervous system. *Electronics*.

[136] Dubový P., Jančálek R., Kubek T., Geuna S., Perroteau I., Tos P., Battiston B. (2013). Chapter seven - role of inflammation and cytokines in peripheral nerve regeneration. *International Review of Neurobiology, Academic Press2013*.

[137] Lee C. Y.-P., Chooi W. H., Ng S.-Y., Chew S. Y. (2023). Modulating neuroinflammation through molecular, cellular and biomaterial‐based approaches to treat spinal cord injury. *Bioengineering & Translational Medicine*.

[138] Pan D., Hunter D. A., Schellhardt L. T cells modulate IL-4 expression by eosinophil recruitment within decellularized scaffolds to repair nerve defects. *Electronics*.

[139] Tang X., Li Q., Huang T. Regenerative role of T cells in nerve repair and functional recovery. *Electronics*.

[140] Michon J., Amend P., Merle M., Samii M. (1990). Microsurgical repair of peripheral nerve lesions: a study of 150 injuries of the median and ulnar nerves. *Peripheral Nerve Lesions*.

[141] Karamian B. A.-O., Siegel N., Nourie B. The role of electrical stimulation for rehabilitation and regeneration after spinal cord injury. *Electronics*.

[142] Jin M. Y., Weaver T. E., Farris A., Gupta M., Abd-Elsayed A. (2023). Neuromodulation for peripheral nerve regeneration: systematic review of mechanisms and in vivo highlights. *Biomedicines*.

[143] Char S., Jin M. Y., Francio V. T. (2022). Implantable peripheral nerve stimulation for peripheral neuropathic pain: a systematic review of prospective studies. *Biomedicines*.

[144] Strand N. A.-O., D’Souza R. A.-O., Hagedorn J. A.-O. Evidence-based clinical guidelines from the American society of pain and neuroscience for the use of implantable peripheral nerve stimulation in the treatment of chronic pain. *Journal of Pain Research*.

[145] Klodmann J., Schlenk C., Hellings-Kuß A. (2021). An introduction to robotically assisted surgical systems: current developments and focus areas of research. *Current Robotics Reports*.

[146] Goh E. Z., Ali T. (2022). Robotic surgery: an evolution in practice. *Journal of Surgical Protocols and Research Methodologies*.

[147] Rodriguez y Baena F., Davies B. (2010). Robotic surgery: from autonomous systems to intelligent tools. *Robotica*.

[148] Buccelli S., Tessari F., Fanin F. (2022). A gravity-compensated upper-limb exoskeleton for functional rehabilitation of the shoulder complex. *Applied Sciences*.

[149] Ranzani R. A.-O., Lambercy O., Metzger J. C. Neurocognitive robot-assisted rehabilitation of hand function: a randomized control trial on motor recovery in subacute stroke. *Electronics*.

[150] Roche A. D., Rehbaum H., Farina D., Aszmann O. C. (2014). Prosthetic myoelectric control strategies: a clinical perspective. *Current Surgery Reports*.

[151] Parker B. J., Rhodes D. I., O’Brien C. M., Rodda A. E., Cameron N. R. (2021). Nerve guidance conduit development for primary treatment of peripheral nerve transection injuries: a commercial perspective. *Acta Biomaterialia*.

